# Flocculation and Dewatering Mechanisms of Pyrite Flotation Tailings: Synergistic Roles of Polyferric Chloride, Polydiallyldimethylammonium Chloride, and Skeleton Builders on Microstructure Evolution

**DOI:** 10.3390/ma19153298

**Published:** 2026-08-04

**Authors:** Hongwei He, Zhuo Liu, Junnan Fan, Xuke Dai, Xuquan Huang, Jun Wang, Xiaorong Zhao, Haojie Wang, Fei Xue, Yuwei Xiang

**Affiliations:** 1 College of Hydraulic & Environmental Engineering, China Three Gorges University, Yichang 443002, China; 2 The Seventh Geological Brigade of Hubei Geological Bureau, Yichang 443002, China; 3 Hubei Provincial Ecological and Environmental Monitoring Center Station, Wuhan 430071, China; 4 Engineering Research Center of Eco-Environment in Three Gorges Reservoir Region, Ministry of Education, China Three Gorges University, Yichang 443002, China; 5 Yichang Key Laboratory of Solid Waste Disposal and Resource Utilization, China Three Gorges University, Yichang 443002, China

**Keywords:** pyrite tailings, solid–liquid separation, fly ash, skeleton builder, microstructure

## Abstract

This study investigates the conditioning and deep dewatering of pyrite tailings slurry using a composite system comprising polyferric chloride (PFC), polydiallyldimethylammonium chloride (PDMDAAC), and fly ash as a skeleton builder. The synergistic application significantly enhanced solid–liquid separation, reducing the filter cake moisture content to 53.6% and the capillary suction time (CST) to 15.1 s. Based on settling and dewatering kinetics, the optimal dosages were established as 5 g/L PFC, 10 mL/L PDMDAAC, and 20 g/L fly ash. Characterization via SEM, XPS, and FTIR elucidated the underlying mechanisms: PDMDAAC facilitated fine particle aggregation through charge neutralization and adsorption bridging, while PFC hydrolysis products reinforced the floc architecture via hydroxyl complexation and sweep flocculation. Crucially, fly ash constructed a robust skeletal framework with efficient drainage channels within the filter cake, effectively mitigating pore clogging. This study provides a high-performance conditioning strategy facilitating the resource utilization of pyrite tailings.

## 1. Introduction

Pyrite, as a principal sulfur resource in China, serves as a fundamental raw material for sulfuric acid production and non-ferrous metal smelting [1]. Driven by the national imperatives of “Zero-Waste City” initiatives and “Dual Carbon” targets, the reduction, valorization, and harmless disposal of mining solid waste have become paramount [2]. Consequently, the safe and efficient management of pyrite tailings—a bulk solid residue generated from beneficiation—represents a critical bottleneck limiting both environmental sustainability and resource recycling [3,4].

Deep dewatering is a prerequisite for subsequent safe landfilling, preparation of building materials, or metal recovery from pyrite-rich slurries [5]. However, the inherent physicochemical characteristics of these tailings, specifically their high density and colloidal stability, pose significant challenges to conventional solid–liquid separation processes. While flocculant-skeleton builder systems have been widely adopted in municipal sludge treatment, their application in sulfide-rich mineral processing residues remains underexplored, particularly regarding the underlying synergistic mechanisms governing floc microstructural evolution.

Compared to conventional polyacrylamides (PAM), polydiallyldimethyl- ammonium chloride (PDMDAAC) offers distinct advantages, including environmental compatibility and low toxicity [6,7,8]. As a linear cationic polyelectrolyte, PDMDAAC is characterized by high charge density and excellent water solubility. Its hybridization with inorganic coagulants—such as polyaluminum chloride (PAC) and polyferric sulfate (PFS)—has proven effective in potable water treatment [6,9]. For instance, Shen et al. [10] demonstrated that PAC-PDMDAAC composites effectively remove natural organic matter (NOM) from surface water. Similarly, composite coagulants consisting of PDMDAAC blended with PAC or PFC have exhibited superior treatment performance compared with conventional inorganic coagulants for various source waters including highly turbid Yellow River raw water to lightly contaminated reservoir water [7,11,12,13]. Liu et al. [14] further highlighted the synergy between PDMDAAC and PAM in coal slurry treatment via charge neutralization and floc structure modulation. Despite these advances, the application of PDMDAAC in the conditioning of pyrite mine tailings remains absent.

A major obstacle in dewatering is the compressibility of flocs formed by organic polymers. Under mechanical pressure, the soft floc structure tends to collapse, leading to the blockage of drainage pathways and a decline in dewatering efficiency [15]. To mitigate this, rigid skeleton builders—such as fly ash or quartz sand—are introduced to construct a porous architecture within the filter cake [16]. Studies have shown that combining flocculants with mineral additives significantly reduces sludge moisture content; for example, Xin et al. [17] reduced sludge water content to 76.1% by integrating quartz sand and fly ash. Zhang et al. [18] confirmed that the combination of SDS and PDMDAAC enhances dewatering performance compared to single agents. Despite these advances, the application of PDMDAAC in conjunction with rigid skeleton builders for the conditioning of sulfide-rich tailings remains largely unexplored.

In this study, we propose a novel conditioning strategy utilizing PFC as the primary coagulant, PDMDAAC as the cationic aid, and fly ash as the skeleton builder. The optimal dosages were determined through systematic settling and dewatering assays. Furthermore, the morphological evolution and chemical interactions within the sludge matrix were characterized using Scanning Electron Microscopy (SEM), X-ray Photoelectron Spectroscopy (XPS), and Fourier Transform Infrared Spectroscopy (FTIR) to elucidate the deep dewatering mechanisms and provide theoretical support for the resource utilization of pyrite tailings.

## 2. Materials and Methods

### 2.1. Experimental Materials

All chemical reagents employed in this study were of analytical reagent grade. Sodium hydroxide (NaOH) and PDMDAAC were procured from Sinopharm Chemical Reagent Co., Ltd. (Yichang Ciy, China). The PDMDAAC product from Shanghai Macklin Biochemical Technology Co., Ltd., had a weight-average molecular weight of 200,000–350,000 and an active-solids content of 20 wt.%. PFC was supplied by Sinopharm Chemical Reagent Co. and had a total iron content of 28 wt.%. Carbide slag, utilized as an alkaline regulator, was obtained from Hubei Yihua Group Co., Ltd.; its primary chemical constituents are detailed in the Supporting Information (Table S1). Fly ash, used as the skeleton builder, was commercially sourced; its oxide composition is presented in Table 1.

The pyrite tailings slurry was collected from the discharge outlet of an abandoned pyrite mine pit in Yichang City, Hubei Province, China. Upon collection, the wastewater was stored at 4 °C to preserve its physicochemical stability prior to testing. The key characteristics of the raw slurry, including pH, sulfate concentration, total metal content, and turbidity, are summarized in Table 2.

### 2.2. Experimental Procedure

The raw pyrite tailing slurry was initially pre-treated with carbide slag to neutralize acidity and adjust the pH to near-neutral conditions. Subsequently, a two-stage conditioning process was implemented using PFC and PDMDAAC.

(1) Single-agent conditioning:

Predetermined dosages of PFC or PDMDAAC were individually introduced into 500 mL of slurry. The mixture underwent rapid mixing at 300 rpm for 3 min, followed by slow stirring at 200 rpm for 15 min to ensure homogeneous flocculation.

(2) Combined conditioning with skeleton builders:

Optimal dosages of PFC and PDMDAAC were sequentially added under rapid mixing (300 rpm). Subsequently, a predetermined dosage of fly ash was introduced as a skeleton builder. The system was then subjected to slow stirring at 200 rpm for 15 min to facilitate the formation of a reinforced floc structure.

### 2.3. Sludge Settleability Assessment

The settleability of conditioned sludge was evaluated using the Sludge Volume Index over a 60-min settling test. Following the flocculation, the mixture was transferred to a 1-L graduated cylinder. The volume of settled sludge was recorded at specific intervals. The Sludge Settling Ratio (SV_60_), defined as the percentage of settled sludge volume after 60 min relative to the initial volume, was calculated to quantify sedimentation performance.

### 2.4. Capillary Suction Time (CST) and Moisture Content Measurement

The dewaterability of the conditioned sludge was assessed using CST and moisture content measurements:

**CST: CST** was measured using a standard CST apparatus (DFC-10A). A representative sludge sample of 5–6 mL was placed on the filter paper, and the time required for water to travel a fixed distance (as indicated by the instrument) was recorded. Lower CST values indicate enhanced dewaterability and faster free water release.

**Moisture content**: Conditioned sludge was dewatered using a laboratory filter press with an effective filtration area of 63.62 cm^2^ and a chamber volume of 250 mL. Conditioned sludge was dewatered under a constant pressure of 0.4 MPa using a laboratory filter press for 30 min. The resulting filter cake was weighed immediately (*m*_2_) and subsequently dried in an oven at 105 °C for 2 h until a constant weight was achieved (*m*_1_). The moisture content (MC) was calculated according to Equation (1):

(1) $$MC=\frac{m_{2}-m_{1}}{m_{2}-m_{0}}\times 100\%$$

where *m*_0_ is the mass of the empty weighing bottle (g); *m*_1_ is the total mass of the dry sample and the weighing bottle (g); and *m*_2_ is the total mass of the wet sample and the weighing bottle (g).

### 2.5. Statistical Analysis

All settling, CST, filter cake moisture content, and pressure filtration experiments were performed independently in triplicate. Quantitative data are presented as the mean ± standard deviation (SD). Statistical analyses were conducted using IBM SPSS Statistics (Version 24.0). One-way analysis of variance (ANOVA) followed by Tukey’s post hoc test was employed to determine significant differences among groups. A *p*-value less than 0.05 (*p* < 0.05) was considered statistically significant.

## 3. Result and Discussion

### 3.1. Effect of Chemical Conditioning on Sedimentation and Dewatering Performance

#### 3.1.1. Effect of PDMDAAC Dosage on the Sedimentation and Dewatering Process

**Figure 1:**
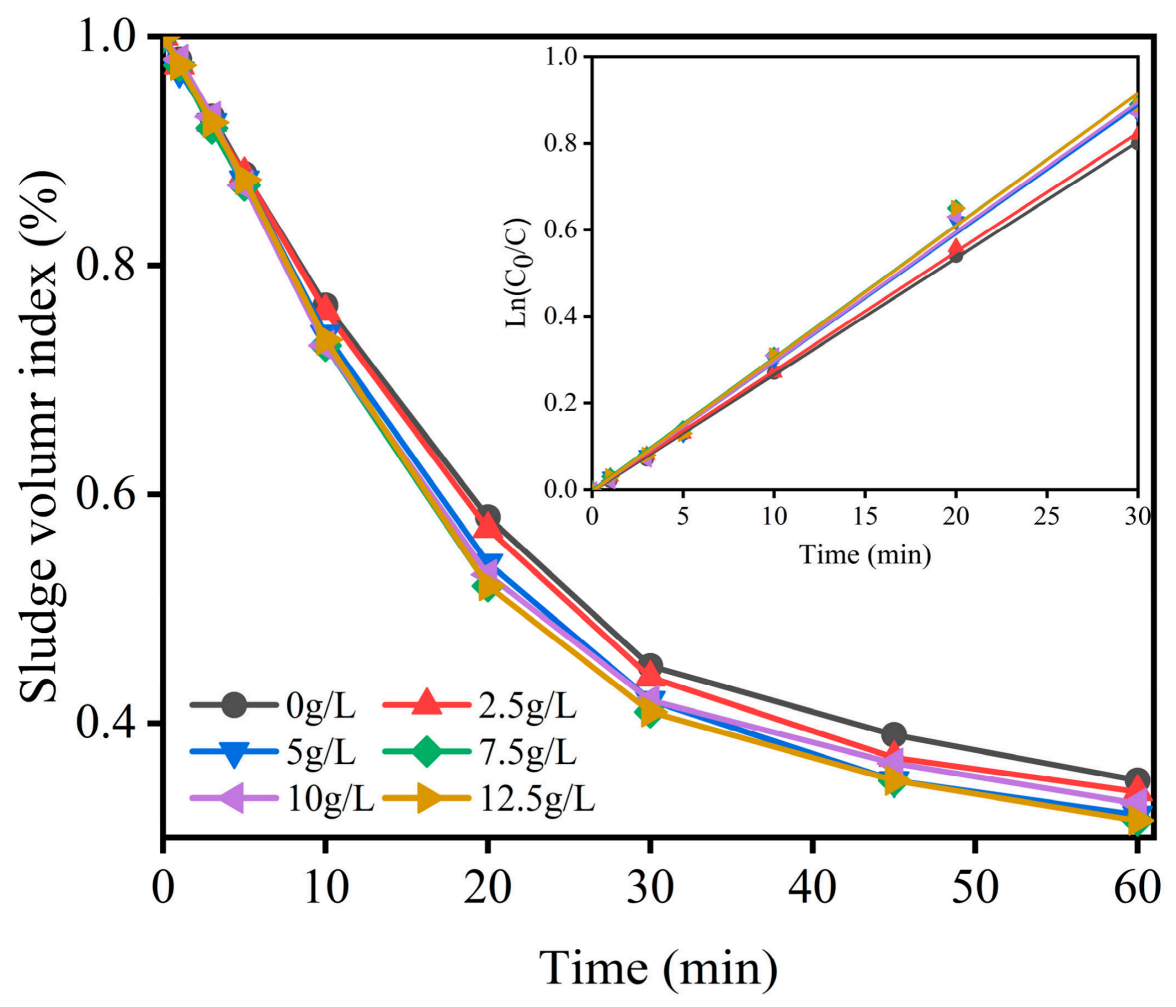
Effect of PDMDAAC dosage on the sedimentation process.

Based on preliminary results, the pyrite tailings slurry was pre-neutralized with 20.0 g/L of calcium carbide slag and conditioned with 10.0 g/L of PFC as the primary flocculant. Figure 1 illustrates the effect of PDMDAAC dosage on the sedimentation kinetics. Clear supernatants were observed under all conditions. The Sludge Settling Ratio (SV) decreased initially before increasing at higher dosages, with the minimum SV recorded at 5.0 mL/L. Kinetic analysis via a first-order model (inset of Figure 1) revealed that the apparent rate constant (*k*) peaked at 5.0 mL/L (*k* = 0.054 min^−1^, R^2^ = 0.9891), confirming optimal flocculation efficiency.

**Figure 2:**
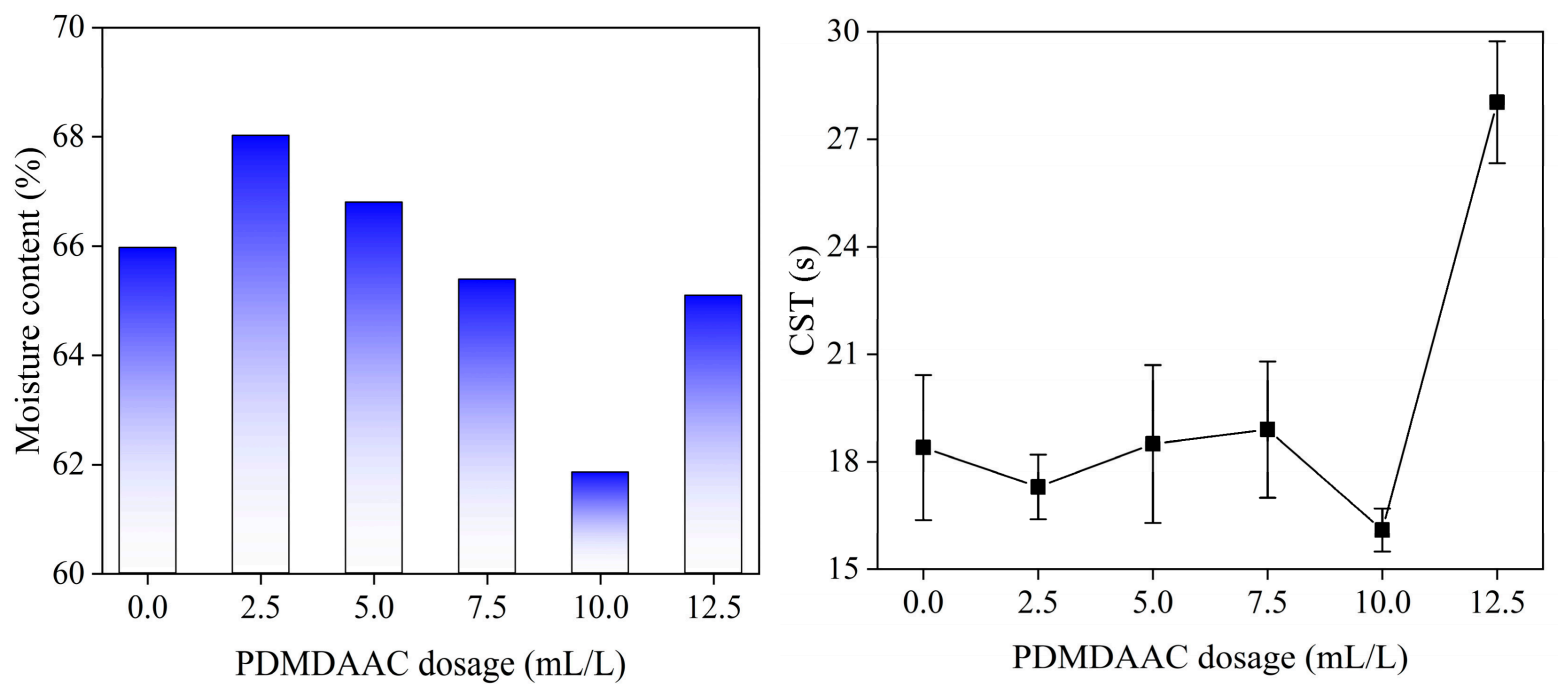
Effect of PDMDAAC dosage on the dewatering performance.

Figure 2 depicts the influence of PDMDAAC dosage on dewaterability. The filter cake moisture content decreased to a minimum of 61.9% at 10.0 mL/L before increasing. Microscopically, lower dosages yielded compact flocs, whereas overdosing (12.5 mL/L) resulted in loose, bulky structures. These voluminous flocs encapsulated substantial interstitial water, which resisted expulsion during pressure filtration [13,14,19]. Correspondingly, the CST reached a minimum of 16.1 s at 10.0 mL/L but surged to 28 s at 12.5 mL/L, indicating that overdosing hindered the release of free water [8,18]. Thus, 10.0 mL/L was identified as the optimum dosage.

#### 3.1.2. Effect of PFC Dosage on the Sedimentation and Dewatering Process

**Figure 3:**
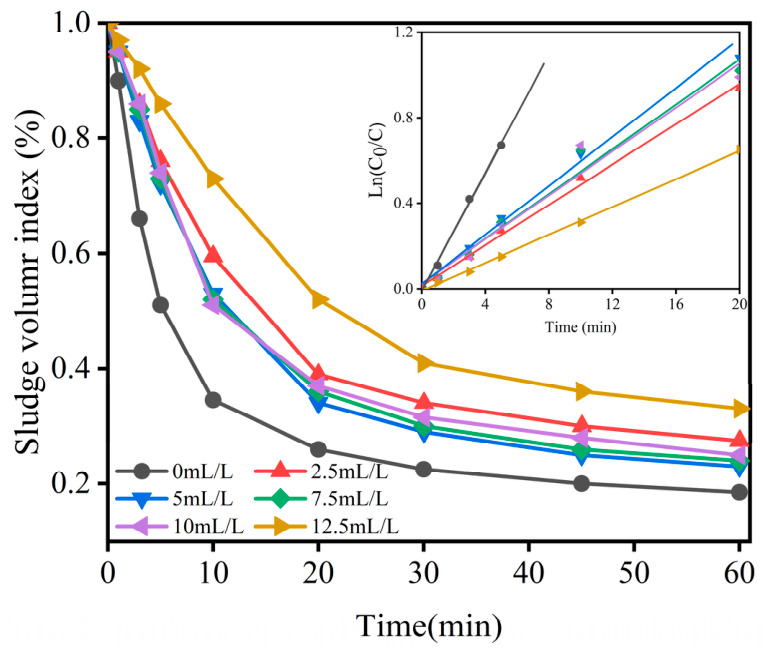
Effect of PFC dosage on the sedimentation process.

Figure 3 illustrates the effect of PFC dosage on flocculation and sedimentation. In the presence of PDMDAAC (10 mL/L), supernatant clarity improved progressively with increasing PFC dosage, attributed to the enhanced charge neutralization of negatively charged pyrite colloids by Fe^3+^ hydrolysis products (e.g., Fe(OH)^2+^, Fe(OH)_2_^+^) [20]. The floc compressibility initially increased (peaking at 5 g/L) before decreasing, aligning with the transition from “sweep flocculation” (dense, compressible flocs) to “excess coagulant” (loose, gelatinous precipitates). The sedimentation rate peaked at 5 g/L PFC, corresponding to the optimal balance between floc size and density.

**Figure 4:**
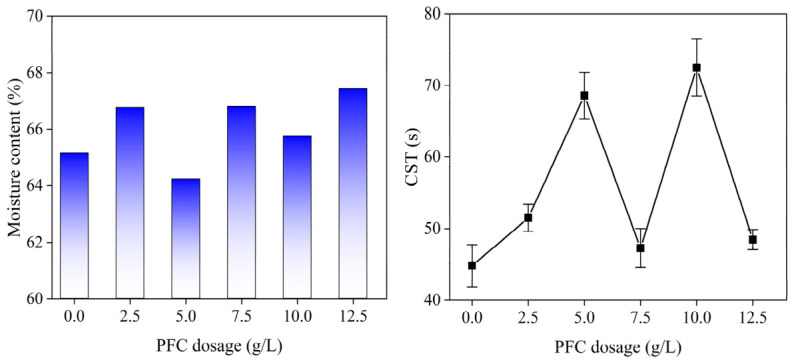
Effect of PFC dosage on the dewatering performance.

Figure 4 presents the influence of PFC dosage on dewatering performance. The moisture content exhibited a non-monotonic trend, reaching a minimum of 64.2% at 5 g/L. This optimum aligns with the formation of densely packed flocs via hydroxyl complexation (Fe^3+^ bridging between particles) and sweep flocculation (enmeshment of fines by amorphous Fe(OH)_3_ precipitates) [21]. Beyond 5 g/L, moisture content increased sharply (e.g., 68.5% at 10 g/L), driven by two mechanisms: (1) Excessive Fe^3+^ hydrolysis generates highly hydrated Fe(OH)_3_ gels that encapsulate water molecules, increasing bound water content [22]. (2) Overdosing leads to “colloid restabilization,” where excess positive charges on flocs cause electrostatic repulsion, preventing tight packing [23].

### 3.2. Effect of Combined Chemical Conditioning and Skeleton Builders on Dewatering Performance

As demonstrated, the dual application of PFC and PDMDAAC effectively accelerated sedimentation rate and improved supernatant clarity. However, chemical conditioning alone yielded marginal reductions in filter cake moisture content. This limitation arises because dewatering efficiency under pressure is governed not solely by particle aggregation but critically by the mechanical stability of the floc structure to preserve porosity for water expulsion. Soft flocs formed by organic polymers are susceptible to compressive deformation, leading to pore channel collapse and impeded water migration [16,17,18].

**Figure 5:**
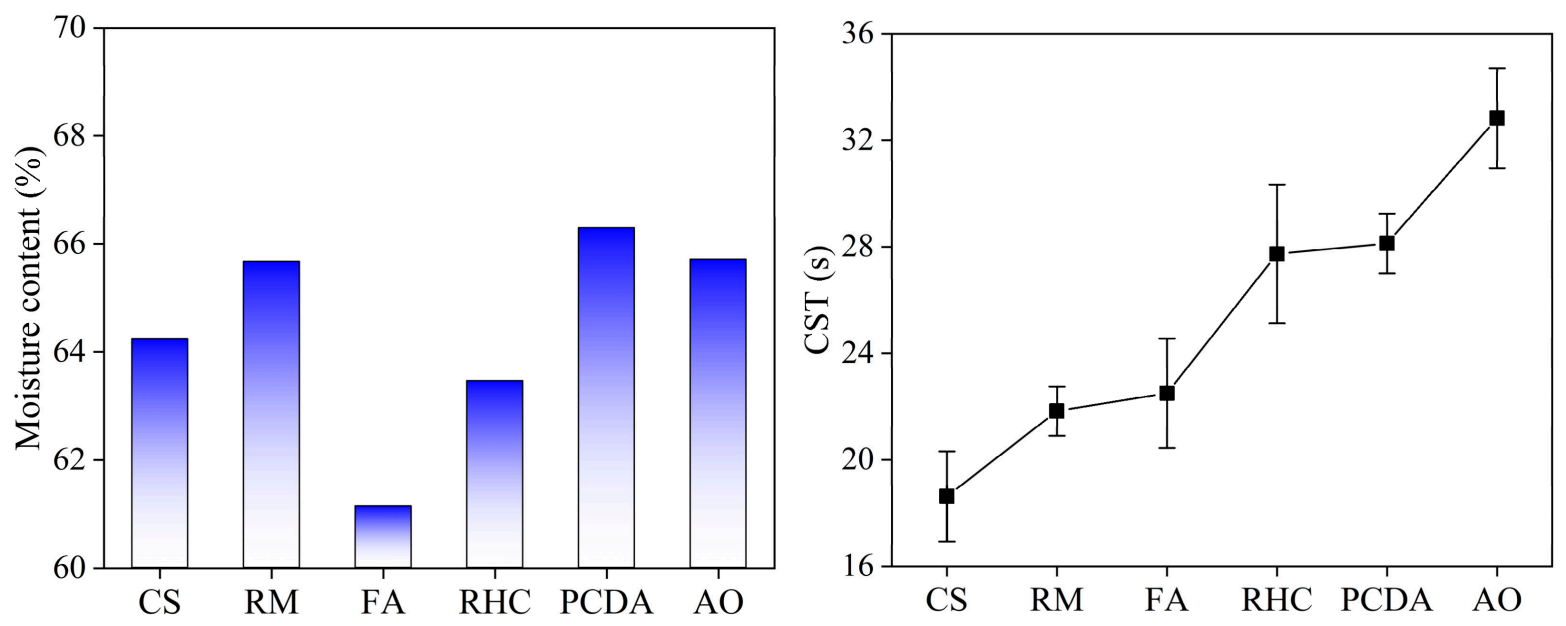
Effect of various skeleton builders on the dewatering process.

To address this, rigid skeleton builders were incorporated to enhance filter cake rigidity and maintain open drainage pathways. Figure 5 compares the dewatering performance of various skeleton builders (dosed at 20 g/L). RM, petroleum coke desulfurization ash, and alumina increased moisture content (65.7–66.3%) compared to the control (64.2%), likely due to their high specific surface area and hydration reactivity, which adsorb water molecules [16]. In contrast, fly ash and rice husk biochar reduced moisture content to 61.1% and 63.4%, respectively. Fly ash’s superiority is attributed to its spherical particle morphology (reducing frictional resistance during filtration) and silica-alumina glassy phase (chemically inert, avoiding additional hydration water) [24]. Rice husk biochar, while effective, introduces organic carbon that may interfere with subsequent pyrite metal recovery, making fly ash the preferred choice for industrial applications.

Compared to sole chemical conditioning, the combined conditioning strategy drastically shortened the CST by 31–75%, underscoring the pivotal role of skeleton builders in facilitating deep dewatering. Consequently, fly ash was selected for further investigation due to its superior performance and potential for large-scale industrial utilization.

**Figure 6:**
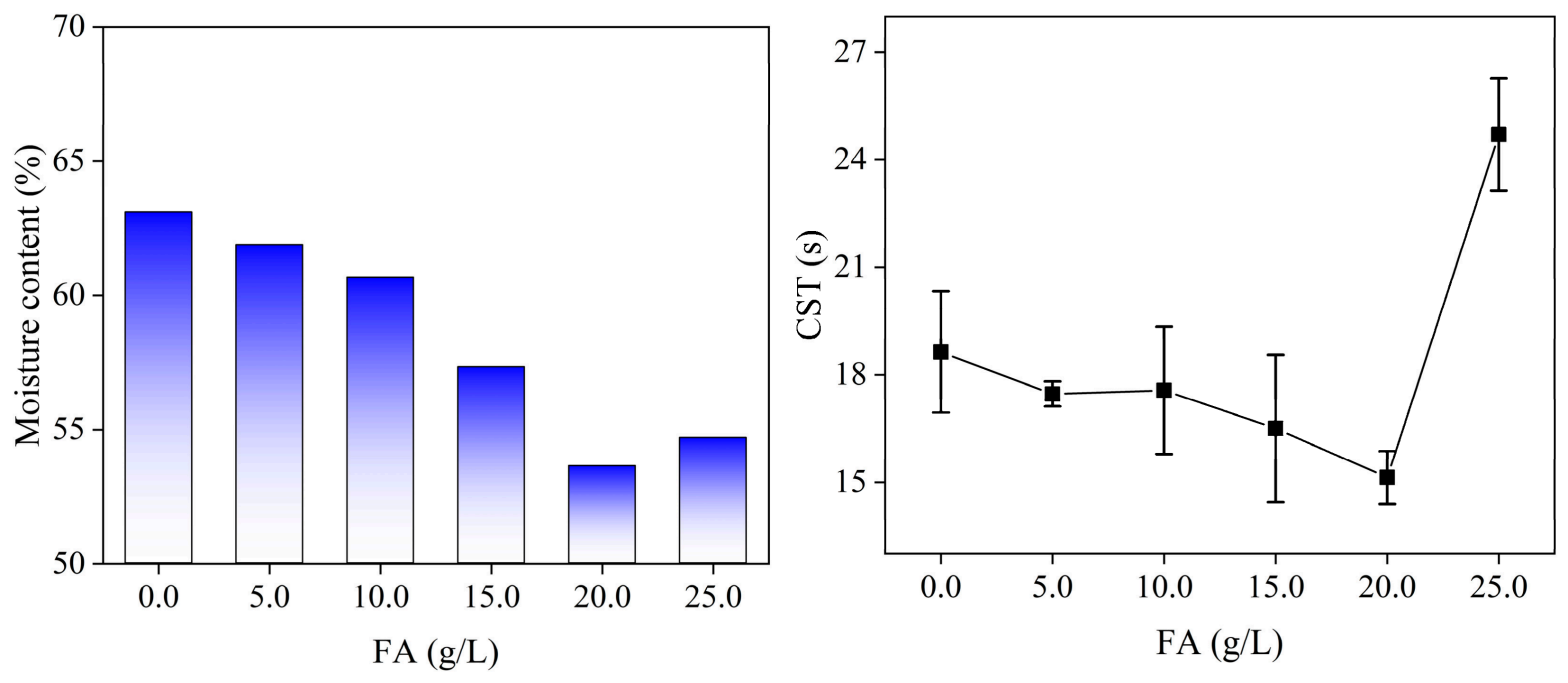
Effect of fly ash dosage on the dewatering process.

Figure 6 depicts the effect of fly ash dosage on dewatering performance. The moisture content exhibited a typical dose–response relationship, reaching a minimum of 53.6% at 20 g/L, following a three-stage mechanism:

- **0–10 g/L**: Insufficient fly ash particles act as isolated “islands,” failing to form a continuous skeletal network. Drainage relies on chemical flocs, with limited improvement (moisture content decreases to 60.7%). The inferior dewatering performance is attributed to the insufficient volume fraction of the skeletal phase, preventing the formation of an interconnected drainage network.
- **10–20 g/L**: A **percolating skeletal network** forms [25], where rigid fly ash particles act as micro-bearings, propping open pores and providing structural support against compressive stress, which allows water to be expelled smoothly along the channels. This “rigid scaffold” resists compressive stress, maintaining high hydraulic conductivity (CST drops to 15.1 s).
- **>20 g/L**: Excess fly ash fills pore spaces, acting as “fine fillers”. These surplus particles not only compete for pore volume but also detach loosely bound fine particles, causing them to migrate and clog the established drainage pathways. This ‘overloading’ effect disrupts the skeletal network continuity, leading to a sharp increase in hydraulic resistance. Hydraulic resistance increases, raising CST to 24.7 s (+63.6%) and moisture content to 54.7%. This deterioration is due to the excess fine particles physically blocking the established drainage pathways, increasing the hydraulic resistance to flow. This “overloading” effect caused the CST to spike and the moisture content to rise by approximately 1.0%.

Based on the above analysis, incorporating 20 g/L of fly ash facilitates the formation of a comprehensive internal skeletal framework. Notably, this structure minimizes reducing dewatering resistance and achieves 53.6% moisture content, approaching the moisture range generally required for CPB feedstock preparation [26], demonstrating the potential relevance of this conditioning strategy for sustainable tailings management.

Although fly ash demonstrated optimal dewatering performance among the assessed conditioners, its utilization is predicated on a thorough understanding of its geochemical heterogeneity. The major-oxide composition (Table 1) serves merely as a bulk indicator and fails to capture the speciation or leachability of potentially toxic trace elements (e.g., As, Pb, Cd, Cr, Hg). Therefore, we explicitly caution against assuming environmental benignity based solely on these data. Future field applications demand rigorous, source-specific characterization coupled with standardized leaching protocols to mitigate the risk of secondary contamination.

## 4. Mechanism Analysis

### 4.1. Microstructural Evolution and Skeletal Support Effect (SEM)

**Figure 7:**
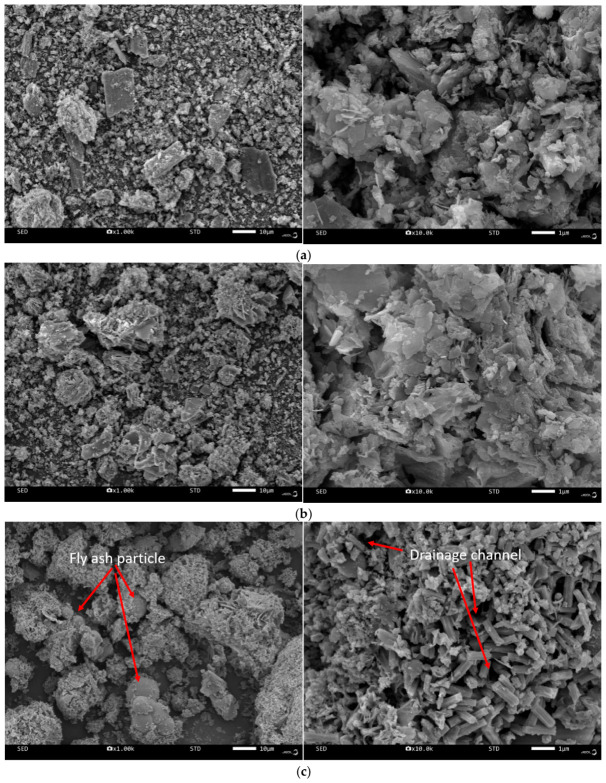
SEM micrographs of filter cake powders: (**a**) Pyrite sediment. (**b**) Pyrite sediment + PFC + PDMDAAC. (**c**) Pyrite sediment + PFC + PDMDAAC + Fly ash.

The microstructural transformation of the filter cakes was characterized via SEM, as presented in Figure 7. The morphology directly explains the variations in dewaterability observed in Section 3.

Figure 7 displays the micrographs of sediments precipitated after treatment with calcium carbide slag, calcium carbide slag + PFC, and calcium carbide slag + PFC + fly ash + PDMDAAC, followed by dewatering, drying, and grinding. Figure 7a shows the sediment treated solely with calcium carbide slag. At 10 μm magnification, the particles exhibited a heterogeneous distribution, comprising dense, large agglomerates and numerous small sheet-like particles. The absence of a porous network results in severe pore channel collapse under 0.4 MPa pressure filtration. This observation aligns with the poor sedimentation performance noted previously. The absence of a skeleton structure resulted in severe compaction of the flocs under pressure filtration, leading to pore channel collapse and water entrapment within the cake, thereby sustaining a high moisture content. At 1 μm magnification, the smooth surfaces of the large particles indicate limited interaction between fines, confirming that alkaline neutralization alone cannot achieve effective solid–liquid separation.

With the introduction of PFC and PDMDAAC (Figure 7b), the particle size increases significantly due to charge neutralization and adsorption bridging. This is attributed to the charge neutralization and double-layer compression effects of PFC, which promoted the cohesion of small particles into larger aggregates. However, the microstructure appears relatively compact but lacks rigidity. The flocs, composed mainly of soft hydrated iron hydroxides, are prone to plastic deformation under pressure, which restricts the release of interstitial water despite the increased floc size.

The most profound change occurs in the full composite system (calcium carbide slag + PFC + PDMDAAC + fly ash) (Figure 7c). At 10 μm magnification, the incorporation of fly ash creates a rough, irregular topography characterized by prominent protrusions and abundant macropores. Crucially, at 1 μm magnification, fly ash particles act as “micro-bearings” embedded within the floc matrix. They form a rigid skeletal framework that props open the pore structure, preventing collapse during filtration.

Crucially, the synergy between PDMDAAC and fly ash governs the formation of these drainage channels. While PDMDAAC effectively initiates flocculation via charge neutralization and polymer bridging, the resulting flocs are predominantly composed of soft, hydrated iron hydroxides. Under the applied pressure of 0.4 MPa, these organic–inorganic flocs exhibit high compressibility, leading to the occlusion of interstitial pores and the obstruction of water migration pathways (Figure 7b). In contrast, the incorporation of fly ash fundamentally alters the mechanical integrity of the filter cake. The rigid, spherical fly ash particles act as “micro-bearings” embedded within the floc matrix. They bear the majority of the compressive load, preserving the interconnectivity of macropores and preventing the soft flocs from undergoing plastic deformation. This structural reinforcement transforms the compacted cake into a permeable medium with sustained hydraulic conductivity. Consequently, the skeletal network established by fly ash serves as the primary conduit for water expulsion, complementing the flocculation function of PDMDAAC and underpinning the drastic reduction in CST and moisture content observed experimentally.

### 4.2. Surface Chemistry and Adsorption Mechanisms (XPS)

**Figure 8:**
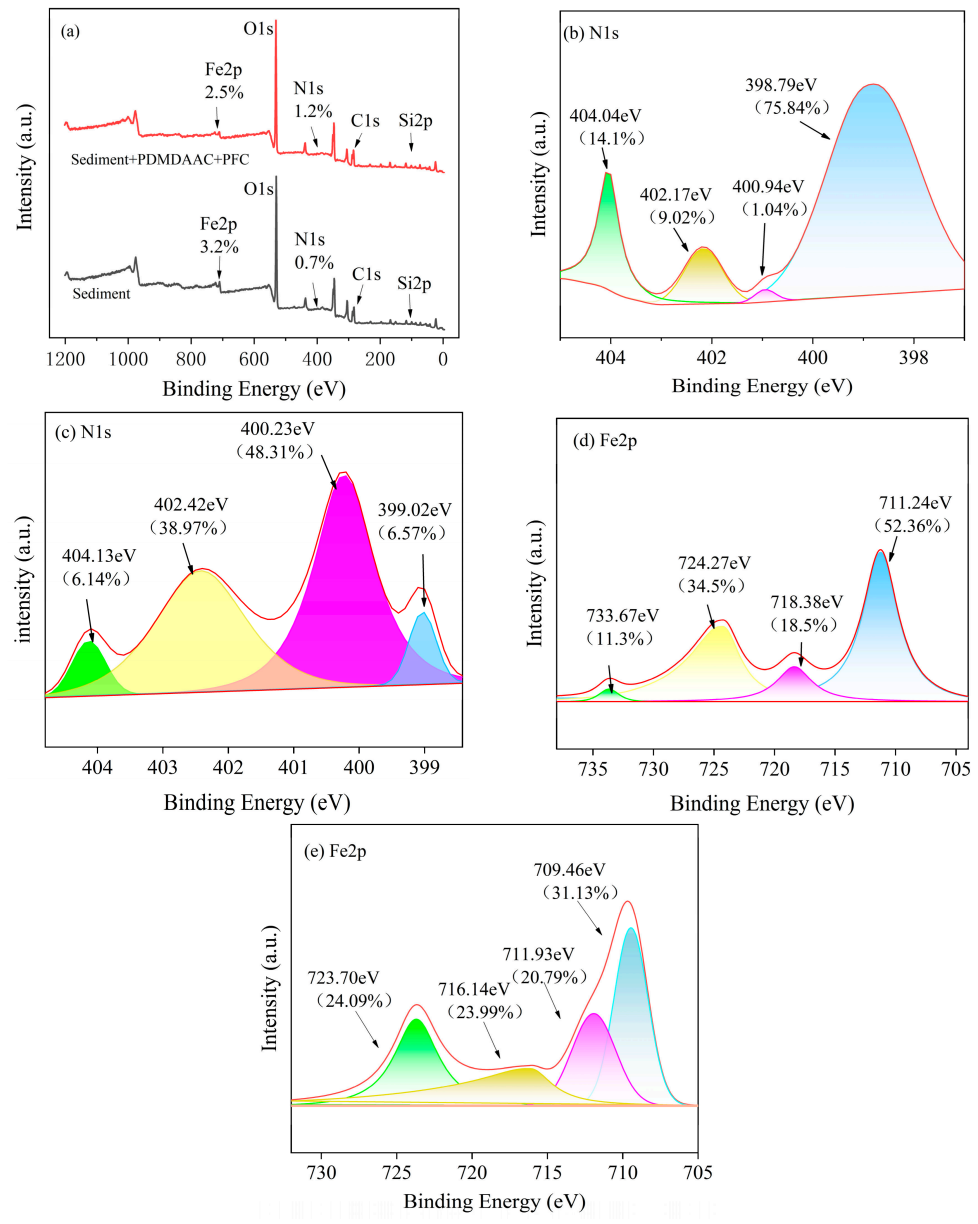
XPS analysis of pyrite sediments: (**a**) Wide-scan survey spectrum; (**b**) N 1s spectrum of raw pyrite sediment; (**c**) N 1s spectrum of sediment treated with PFC and PDMDAAC; (**d**) Fe 2p spectrum of raw pyrite sediment; (**e**) Fe 2p spectrum of sediment treated with PFC and PDMDAAC.

XPS analysis was conducted to elucidate the chemical interactions between the conditioners and the pyrite tailings surface (Figure 8). The wide-scan spectrum (Figure 8a) reveals a distinct N 1s peak in the conditioned sample (1.2%) compared to the raw sediment (0.7%), confirming the successful loading of PDMDAAC.

High-resolution N 1s spectra (Figure 8b,c) further clarify the adsorption mechanism. In the raw sediment, the N 1s signal is negligible. After conditioning, the peak at 402.42 eV (assigned to the quaternary ammonium groups, -N^+^(CH_3_)_3_, of PDMDAAC) increases dramatically, accounting for 39.0% of the total area. Simultaneously, the peak at 400.23 eV (corresponding to the amide groups of PDMDAAC) rises from 1.0% to 48.3%. This quantitative evidence confirms that PDMDAAC adsorbs onto the negatively charged tailings surface via electrostatic attraction [27], where the high density of cationic sites neutralizes surface charges, reducing electrostatic repulsion and enabling floc aggregation, optimizing the subsequent dewatering process.

High-resolution Fe 2p spectra further elucidate the hydrolytic behavior of PFC and its interfacial interactions with the tailings surface (Figure 8d). In the raw tailings, the Fe 2p profile is dominated by signals corresponding to inherent ferric oxides/hydroxides. Following conditioning, a prominent peak emerges at 711.93 eV, assigned to Fe(III)–O/Fe(III)–OH species. This confirms that the hydrolysis products of PFC—specifically amorphous Fe(III) hydroxides—deposit onto or adsorb onto the pyrite particle surfaces. This process alters the surface coordination environment of iron and facilitates the aggregation of fine particles through hydroxyl complexation and sweep flocculation. When correlated with the N 1s results, it is evident that PDMDAAC concurrently reinforces floc architecture via electrostatic patch attraction and polymer bridging. This synergistic mechanism—wherein inorganic hydrolysis products provide densification while the cationic polymer enhances floc connectivity—underpins the enhanced dewatering performance observed experimentally.

### 4.3. Fourier Transform Infrared Spectroscopy (FTIR)

**Figure 9:**
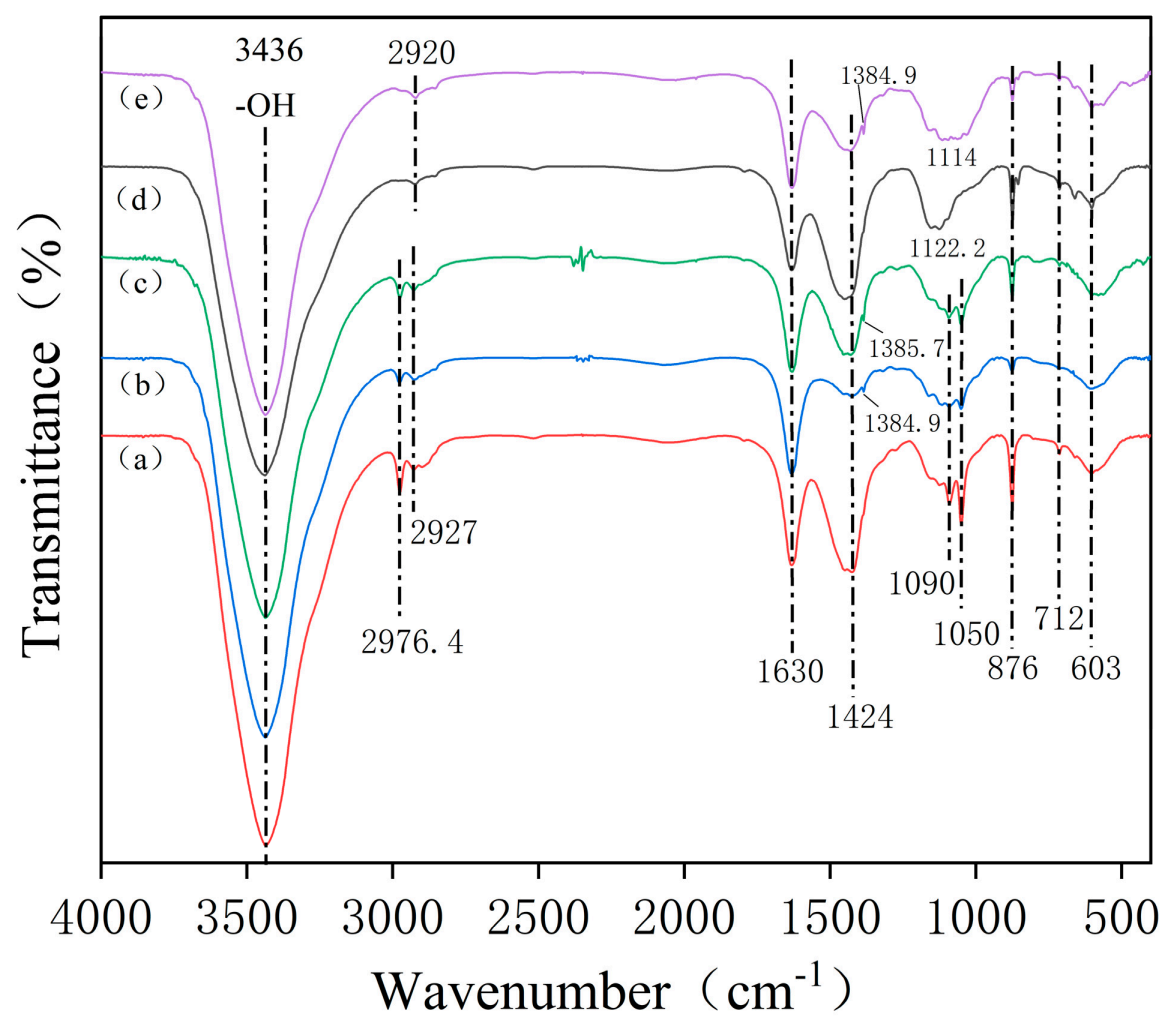
FTIR spectra of pyrite sediments: (**a**) Raw sediment. (**b**) Sediment with PDMDAAC. (**c**) Sediment with PFC. (**d**) Sediment with PDMDAAC and PFC. (**e**) Sediment with PDMDAAC, PFC, and fly ash.

FTIR was employed to identify the functional groups responsible for floc formation (Figure 9). The broad band at 3436 cm^−1^ (O-H stretching) and the peak at 1630 cm^−1^ (O-H bending) confirm the prevalence of iron hydroxides in all samples.

- PDMDAAC treatment (Figure 9b): The appearance of peaks at 2927 cm^−1^ (-CH_2_) and 2976 cm^−1^ (-CH_3_) verifies the adsorption of the polymer. The attenuation of the peak near 876 cm^−1^ (Fe-O-H bending) suggests that PDMDAAC chains coat the inorganic particles, masking the Fe-OH response and altering the surface coordination environment.
- PFC treatment (Figure 9c): The enhancement of the 876 cm^−1^ peak indicates the formation of Fe-O-H bridges. This corresponds to the hydrolysis of PFC and the adhesion of iron hydroxide complexes to the sediment surface via hydroxyl complexation.
- Combined conditioning (Figure 9d): The simultaneous enhancement of C-H (organic) and Fe-OH (inorganic) peaks demonstrates a synergistic effect. The inorganic-organic composites do not merely coexist; they interact to form a denser, more cohesive floc architecture.
- Addition of fly ash (Figure 9e): The broadening of the O-H peak and the diminishment of other peaks suggest a physical dilution effect. Unlike PDMDAAC/PFC, fly ash does not introduce new chemical bonds. Instead, it functions as an inert, rigid filler that modulates the physical pore structure, which aligns perfectly with the SEM observations of skeletal support.

### 4.4. Environmental and Engineering Implications

The significant reduction in filter cake moisture content to 53.6% achieved in this study represents a critical precondition for the downstream resource utilization of pyrite tailings. Notably, this moisture level approaches the reported feedstock requirement for cemented paste backfill (CPB, typically <55%), indicating the potential to convert high-water-content slurry into a transportable solid residue. However, it must be emphasized that the immediate field application of the dewatered tailings as CPB or supplementary cementitious materials is not yet warranted. While the conditioning system optimizes solid–liquid separation, the engineering suitability of the final product depends on unassessed parameters: the unconfined compressive strength (UCS) of the consolidated backfill, long-term structural durability under underground conditions, and the immobilization efficiency of sulfide-associated heavy metals. Comprehensive geotechnical validation is therefore indispensable prior to any mine backfilling implementation.

Beyond performance requirements, the environmental compatibility of the conditioning agents themselves warrants rigorous scrutiny. Fly ash was selected as the skeleton builder herein owing to its superior dewatering efficacy relative to alternative additives. Nevertheless, its chemical composition exhibits marked variability contingent upon its combustion source and coal feedstock. The major-oxide profile presented in Table 1 only reflects bulk mineralogy and fails to capture the speciation or mobility of trace elements of concern—notably As, Pb, Cd, Cr, and Hg. Similarly, the carbide slag used for pH pre-adjustment requires parallel leaching assessment to preclude alkali or heavy metal release. We explicitly caution against assuming environmental benignity based solely on dewatering performance metrics. Future large-scale deployment of this conditioning strategy mandates source-specific total-elemental characterization of all solid additives, coupled with standardized batch leaching tests (e.g., TCLP or EN 12457) to mitigate the risk of secondary contamination. This study thus provides a validated deep-dewatering protocol, while the subsequent environmental and geotechnical validation constitutes a necessary next step for industrial translation.

## 5. Conclusions

This study proposed a novel ternary conditioning strategy utilizing polyferric chloride (PFC), polydiallyldimethylammonium chloride (PDMDAAC), and fly ash for the deep dewatering of pyrite tailings. The key conclusions are as follows:

(1) Optimal Performance: The synergistic system achieved a remarkable reduction in filter cake moisture content to 53.6% and capillary suction time (CST) to 15.1 s under optimal dosages (PFC 5 g/L, PDMDAAC 10 mL/L, fly ash 20 g/L), outperforming single or binary conditioning systems.
(2) Mechanism Elucidation: The synergy originates from the chemical bridging of PFC/PDMDAAC (for aggregation) and the physical bracing of fly ash (for dewatering). PDMDAAC facilitated rapid flocculation via charge neutralization and adsorption bridging, while PFC hydrolysis products enhanced floc density through hydroxyl complexation. Crucially, fly ash acted as a rigid skeleton builder, constructing a porous framework that prevented the collapse of soft flocs under pressure, thereby maintaining open drainage channels.
(3) Resource Implication: The ternary conditioning system reduces the filter cake moisture content to 53.6%, approaching the feedstock requirement for cemented paste backfill (CPB). However, the practical application of the dewatered tailings requires further validation of unconfined compressive strength, long-term durability, and heavy metal leaching characteristics. Coupled with source-specific environmental assessment of fly ash and carbide slag, this strategy provides a viable pathway for the synergistic disposal of pyrite tailings and industrial solid wastes.

## Figures and Tables

**Table 1:** Oxide composition of the fly ash (mass fraction, %).

Composition	SiO_2_	Al_2_O_3_	Fe_2_O_3_	CaO	K_2_O	SO_3_	Others
Mass fraction (%)	54.10	29.88	5.89	3.51	2.45	1.91	2.26

**Table 2:** Physicochemical characteristics of the raw pyrite tailings slurry.

Parameter	pH	Turbidity (NTU)	SS (mg/L)	Fe (mg/L)	Mn (mg/L)	Mg (mg/L)	Ca (mg/L)	SO_4_^2−^ (mg/L)
Value	2.1	0.29	15	517.5	3.821	176.7	325	4830.8

## Data Availability

The original contributions presented in this study are included in the article and Supplementary Material. Further inquiries can be directed to the corresponding author.

## Supplementary Materials

The following supporting information can be downloaded at: https://www.mdpi.com/article/10.3390/ma19153298/s1, Table S1: Major oxide composition of carbide slag (mass fraction, %); Figure S1: Settling characteristics of sludge under different conditioning agents and durations.
