# Supplementary material for: Flocculation and Dewatering Mechanisms of Pyrite Flotation Tailings: Synergistic Roles of Polyferric Chloride, Polydiallyldimethylammonium Chloride, and Skeleton Builders on Microstructure Evolution
Source: Materials (Basel). 2026 Aug 4;19(15):3298. doi: 10.3390/ma19153298 (PMC13467421; doi:10.3390/ma19153298)
Supplement: Supplementary file 1 [file materials-19-03298-s001.zip › materials-4423524-supplementary.pdf]

Carbide slag, employed as an alkali source, was supplied by Hubei Yihua Group Co., Ltd. (China); its oxide composition is summarized in Table S1.

Table S1. Major oxide composition of carbide slag (mass fraction, %).

Composition	CaO	SiO_2	Al_2O_3	Na_2O	SO_3	Fe_2O_3	Others
Mass fraction (%)	93.13	3.01	1.11	0.78	0.43	0.20	1.34

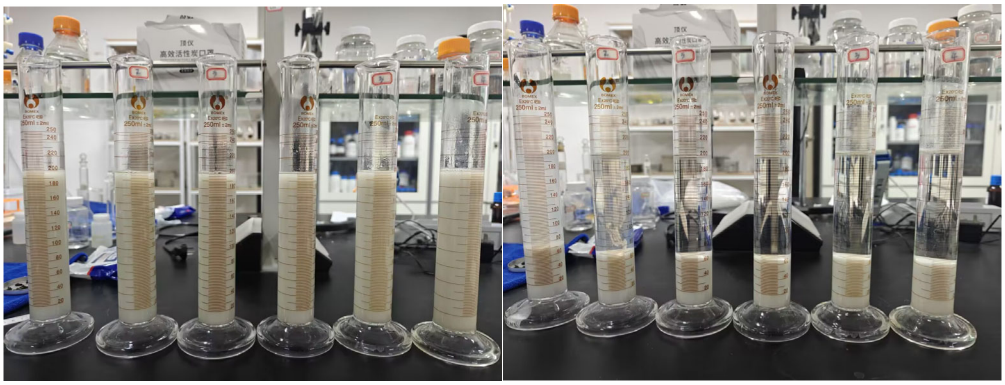

PFC

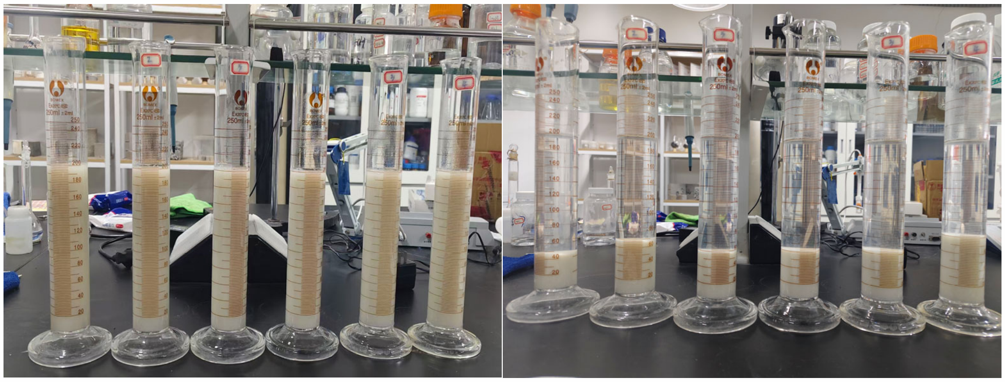

PDMDAAC

Figure S1 Settling characteristics of sludge under different conditioning agents and durations.
